# Implementation of the MoSCoW Method and User-Centered Design to Prioritize Features for a Decision Aid Navigator (DEAN) System: Single-Case Qualitative Process Study

**DOI:** 10.2196/30427

**Published:** 2026-10-23

**Authors:** Brad Morse, Carrie Reale, Erin Latella, Hannah D Bauguess, Lisa M Schilling, Andrey Soares

**Affiliations:** 1 Department of Medicine, Division of General Internal Medicine, University of Colorado Anschutz, 1890 North Revere Court, Suite P12-3200, Aurora, CO, 80045, United States, 1 3037245138; 2 Adult and Child Center for Outcomes Research and Delivery Science, University of Colorado School of Medicine, Aurora, CO, United States; 3 Center for Research and Innovation in Systems Safety, Vanderbilt University Medical Center, Nashville, TN, United States

**Keywords:** user-centered design, MoSCoW method, feature prioritization, decision aids, end-user engagement

## Abstract

**Background:**

User-centered design (UCD) processes often generate extensive lists of potential software features, necessitating prioritization methods to guide development. The MoSCoW method, categorizing features as “must have,” “should have,” “could have,” or “won’t have,” is widely used in software development for its simplicity and ease of adoption. Despite its popularity, limited evidence exists on its application within health informatics. The Decision Aid Navigator (DEAN) system, a clinical decision support tool, required prioritization of features for its administrative portal to ensure usability and alignment with health information technology (HIT) professionals’ needs.

**Objective:**

This study aims to describe how health professionals engaged with the MoSCoW prioritization process during the design of the DEAN administrative portal and to consider how discussion and interaction influenced feature prioritization outcomes.

**Methods:**

A 74-minute web-based group session included 4 HIT professionals experienced in managing clinical decision support systems. Participants independently rated 30 proposed portal features using MoSCoW categories following brief discussions of each feature. Session transcripts, videos, and rating behaviors were analyzed using a single-case qualitative analysis to describe how the MoSCoW process supported prioritization and expert engagement.

**Results:**

Participants rated 37% (11) of 30 features as “must have,” 13% (4) as “should have,” 20% (6) as “could have,” and none as “won’t have.” The remaining 30% (9) showed distributed ratings without a clear majority. The summary percentages are derived from majority vote, not from individual-level ratings. All participants adjusted their ratings, often prompted by group discussion or clarification from the technical lead. Qualitative analysis describes four key insights: (1) interpretations of feature descriptions may vary; (2) group discussion generated suggestions for alternative design solutions; (3) group discussion identified implementation considerations; and (4) participants considered differences in real-world use vs idealized use when ranking features.

**Conclusions:**

The MoSCoW method appeared feasible, accessible, and potentially pragmatic for prioritizing features in a UCD process for a clinical decision support system. Group discussion enriched the prioritization session by surfacing implementation insights and design refinements beyond numeric rankings alone. Findings suggest, in this single case, that the use of MoSCoW is understandable to health informatics teams seeking structured yet flexible stakeholder engagement in software feature prioritization.

## Introduction

User-centered design (UCD) processes often generate many potential software features [1-4], making systematic prioritization essential to balance limited resources with usability goals. Prioritization serves as a key transitional step between user requirements discovery and software implementation, enabling development teams to identify the functionality that should be advanced into prototypes and production systems.

However, when designers engage collaborators to gain insider perspectives, they may encounter a well-documented phenomenon. This phenomenon, often described as a design trap, can arise in both UCD sprints focused on narrowly defined user groups and human-centered design sprints aimed at population-level users. It is known as overreliance [5]. Iteratively interacting with end users early and throughout the design process is a core principle of both UCD and human-centered design. Design solutions emerge not only from discussions with relevant users on how they view and think about the problem, but also in their perspectives on how to solve the problem. These solutions are sometimes described in terms of needs and requirements for the forthcoming health information technology (HIT). Collaborators can often go beyond the scope of a proposed solution in their feature recommendations.

When scope creep is present in the development of HIT as a result of obtaining recommendations from potential users, teams may need processes to narrow the number of proposed needs and requirements. The MoSCoW (must have, should have, could have, or won’t have) method [6-9] is a widely used prioritization approach in software development for categorizing features [10-12]. Despite its widespread use, little research has examined how MoSCoW functions within health informatics UCD workflows or how stakeholder interactions during prioritization shape design decisions. Importantly, successful designs take insights from not only users of the HIT, but also from multidisciplinary teams’ expertise and creative problem solving that those teams have developed.

We applied the MoSCoW method to the development of the administrative portal of the Decision Aid Navigator (DEAN) system [4]. DEAN was co-designed with stakeholders from 4 academic medical centers and aimed to reduce barriers to decision aid use by embedding a provider-facing SMART on FHIR application [13-15] within the electronic health record to support shared decision-making [4]. The administrative portal (Figure 1) enables HIT professionals to manage decision aid metadata and associated clinical quality language logic used to trigger appropriate tools. During the exploratory UCD process, qualitative analysis of technical expert interviews identified 30 candidate features for the portal application.

**Figure 1.**
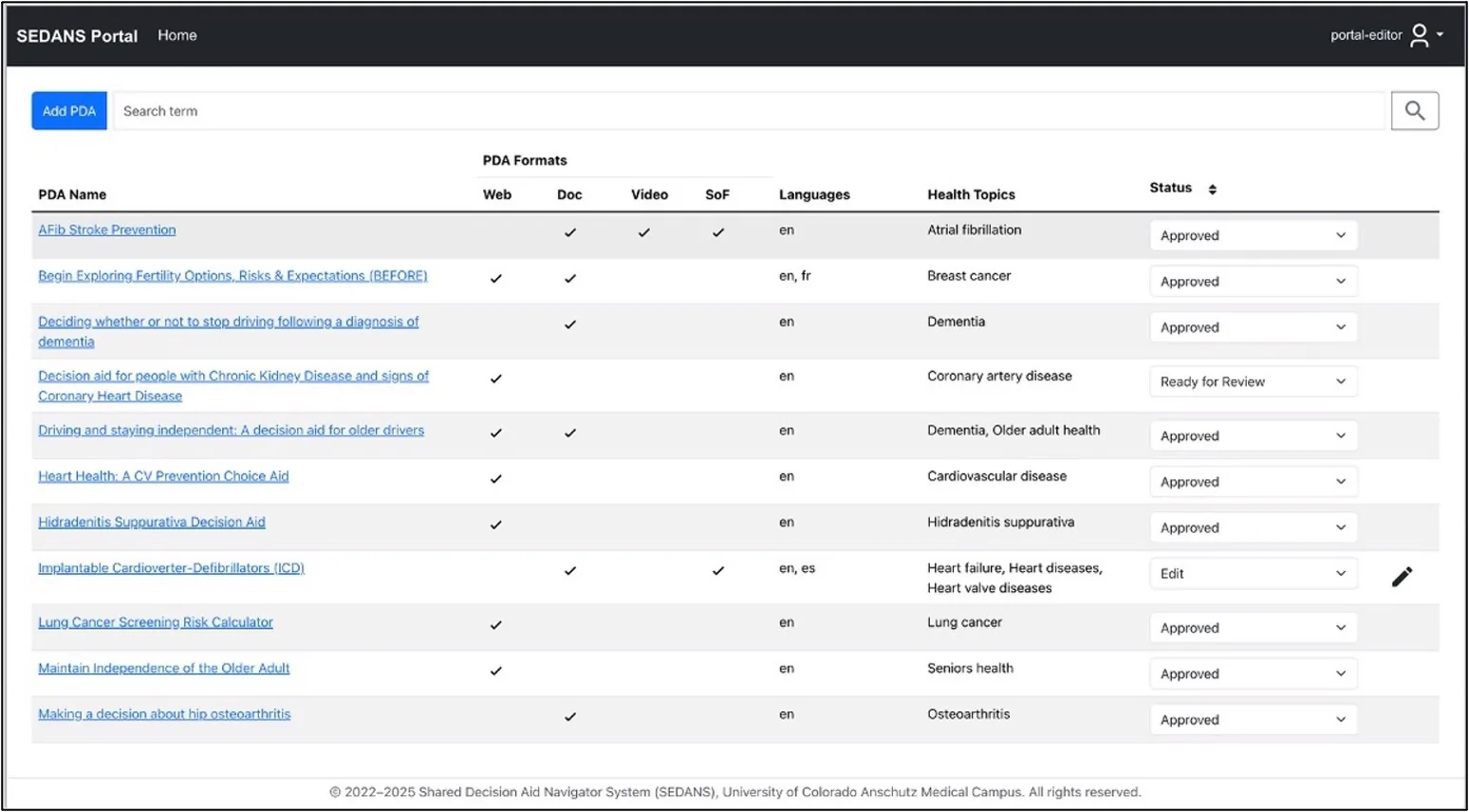
DEAN (Decision Aid Navigator) administrative portal, known as Shared Decision Aid Navigator System (SEDANS) at the time of the MoSCoW (must have, should have, could have, won’t have) prioritization session. AFib: atrial fibrillation; CV: cardiovascular; PDA: patient decision aid.

The MoSCoW method’s main strength is its ability to yield insights with fewer participants. It is easy to learn and apply, enabling project teams to quickly gather meaningful user feedback and identify high-value features [16,17]. This ease of use promotes greater confidence and engagement than more complex prioritization methods [16,17]. However, MoSCoW does not distinguish priorities within categories, which can limit its usefulness for feature-rich projects or cause overuse of the “must have” category [16,18].

Evidence of its implementation in health research, particularly for clarifying priorities and limiting scope creep, remains limited. Although prior studies report widespread use and acceptability, actual application in health research is not well described [10,11,16,18]. This study provides insight on HIT professionals' engagement with the MoSCoW method in a UCD process and makes recommendations on the use of this method.

## Methods

### Procedure

We ran a 74-minute UCD session, using Zoom [3,19,20] and Mural [20-22], with 4 HIT professionals recruited using purposive criteria, including clinical decision support oversight and electronic health record decision authority. Participants had not previously worked together but had completed DEAN discovery interviews.

A human factors facilitator presented the 30 features on 4-quadrant MoSCoW grids (Figure 2). After brief orientation, each feature was presented, discussed (~3 minutes), and independently ranked, followed by further discussion. Features were reranked based on the discussions. If ratings were split, ties were resolved by our research team members that included a clinician, medical informaticians, and research support staff. Decisions on splits were based on multiple considerations, including timelines, cost, dependencies, and overlap with other features.

**Figure 2.**
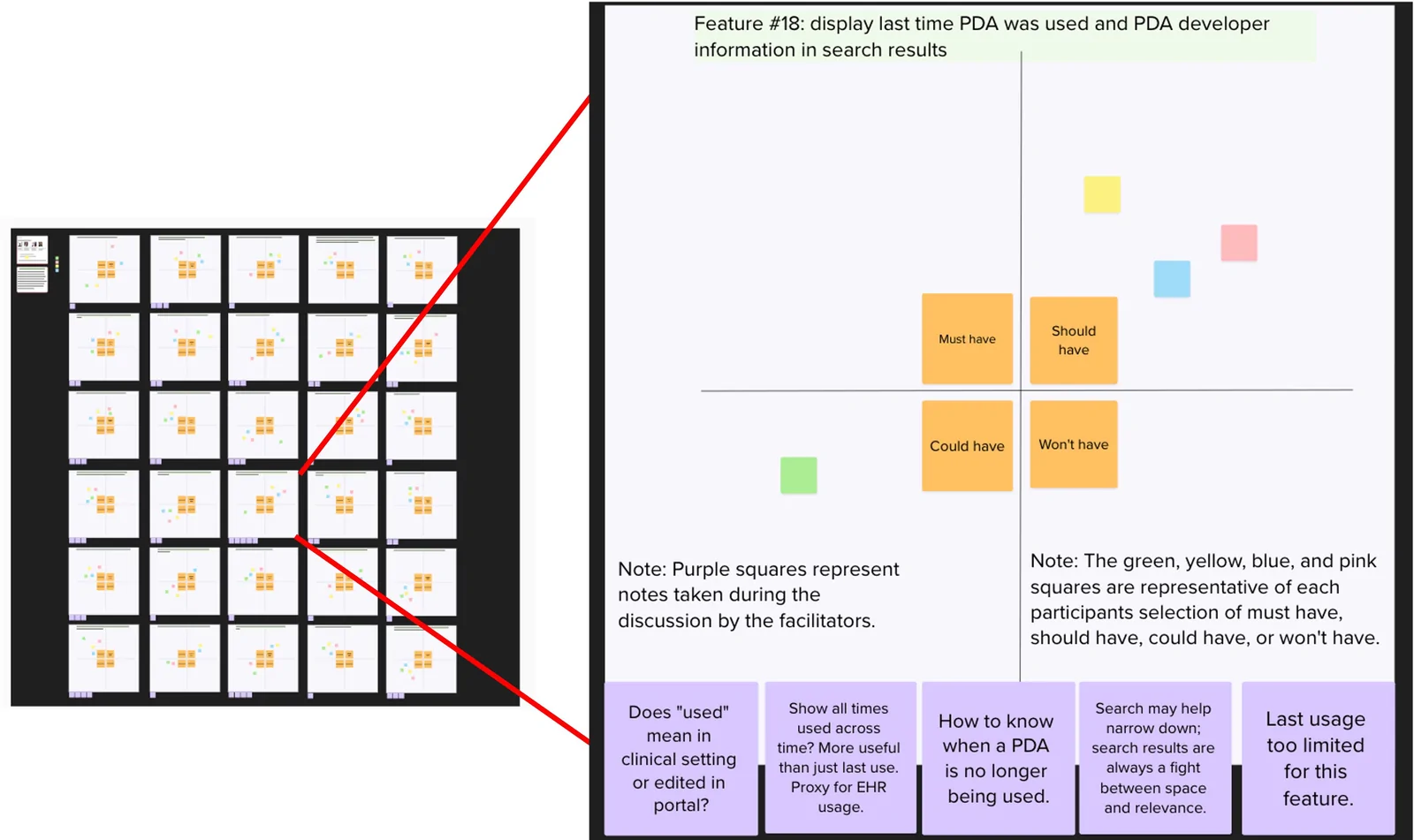
Mural board structure for the MoSCoW (must have, should have, could have, won’t have) prioritization session. EHR: electronic health record; PDA: patient decision aid.

We conducted a single-case qualitative process study [23,24] to examine MoSCoW feature prioritization. The case study approach can be useful when researchers need to obtain a deep appreciation of an interaction in its real-world context [25]. More narrowly, a single-case study was the design selected because it allows evaluation of the MoSCoW process in a bound system that is influenced by hospital-affiliated informatics and HIT development process, restricted budgets, and the requirements of HIT professionals who would ultimately be doing the work in the DEAN administrative portal [26]. Therefore, the single MoSCoW interaction is our unit of analysis. The analysis aims to develop a descriptive explanation of how the MoSCoW method functions within the field of health informatics (ie, how did the interaction take place, what influenced the interaction, and what features were implemented in the DEAN system as a result of the work completed) [27].

Two researchers independently reviewed transcripts and video of the MoSCoW interaction using a descriptive approach [28,29] and rapid qualitative analysis template [30-32], iterating to consensus [33]. We counted each marker placement and any change, defined as moving a marker between quadrants during discussion, and integrated counts with consensus summaries. Nonparticipant attendees included a DEAN technical lead who addressed technical questions and a human factors expert who observed and took notes. Sessions were recorded on Zoom with Mural screen capture and transcribed, and participants received a gift card.

Regarding qualitative reflexivity, our research team—primarily the facilitator of the MoSCoW session—aimed to mitigate power dynamics within the group by selecting participants who were technical leads at different institutions with no prior relationships [34]. Facilitators had no stake in design choices, emphasized independent judgment, and minimized influence via structured, low-intervention facilitation. Furthermore, the facilitator attempted to eliminate any assumptions on what was spoken and utilized in situ member checking [35] to verify statements when needed.

### Ethical Considerations

This study was reviewed and approved by the Colorado Multiple Institutional Review Board (COMIRB 22‐1061). All participants provided informed consent before participating in the web-based prioritization session. Participation was voluntary, and participants could withdraw at any time without penalty. The study involved minimal risk and did not collect protected health information or other sensitive clinical data. Audio recordings, transcripts, and feature prioritization ratings were deidentified prior to analysis and stored on secure, access-controlled institutional servers. All study data were deidentified before analysis, and no information that could reasonably identify individual participants is reported in this manuscript. Participants received a $50 gift card as compensation for their time.

## Results

Four HIT professionals completed the 74-minute group session, representing health system technology and clinical informatics roles. Demographics were as follows: for gender, 3 were male and 1 participant’s gender was not reported; for age, 3 were 41‐49 years and 1 was 50+ years; and for race, 2 were White and 2 participants’ race was not reported.

Participants grasped the feature descriptions (Multimedia Appendix 1) with minimal explanation and immediately began rating. For 28 (93%) of the 30 features, participants started group discussion to clarify feature descriptions for shared understanding, offer feedback, and share valuable sociotechnical advice. Two of the features were ranked without comments or questions.

Table 1 describes the ratings. Participants rated a mean of 12 features before any group discussion began. All participants changed their initial rating more than once (range 3‐6 changes). Participants stated they were changing their rating based on discussion and clarifications (eg, “That’s less important.”). All rating changes occurred for features where group discussion took place.

**Table 1.** Participant ratings and changes on 30 features.

Rating and change type	Features rated by participant, n (%)				Number of features rated, mean (SD)	Total changes in ratings, n (%)
P1	P2	P3	P4
Initial ratings made before any group discussion	14 (47)	10 (33)	9 (30)	15 (50)	12 (2.94)	—^a^
Ratings changed during reflection and discussion period	3 (10)	6 (20)	5 (17)	5 (17)	4.75 (1.26)	19 (63)
Features for which ratings were changed at final rating	3 (10)	5 (17)	4 (13)	5 (17)	4.25 (0.96)	17 (57)
Changed to increase priority	1 (3)	1 (3)	3 (10)	2 (7)	1.75 (0.96)	7 (23)
Changed to decrease priority	2 (7)	5 (17)	2 (7)	3 (10)	3.00 (1.41)	12 (40)
Final rating category						
Must have	14 (47)	11 (37)	12 (40)	12 (40)	12.25 (1.09)	—
Should have	11 (37)	7 (23)	7 (23)	9 (30)	8.5 (1.66)	—
Could have	5 (17)	11 (37)	11 (37)	7 (23)	8.5 (2.60)	—
Won’t have	0 (9)	1^b^ (3)	0 (0)	0 (0)	0.25 (0)	—
Not rated	0 (0)	0 (0)	0 (0)	2^c^ (7)	0.5 (0)	—

a Not applicable.

b P2 classified feature 13 (SME [subject matter expert] details) as “won’t have.” This will not be reflected in summary percentages because, overall, feature 13 was coded as “could have,” based on majority vote.

c P4 did not vote on 2 features based on accessibility during a brief internet outage.

Participants rated 37% (11) of the 30 features as “must have,” 13% (4) as “should have,” 20% (6) as “could have,” and none as “won’t have” (based on majority rules). The remaining 30% (9) of the features had split ratings showing no majority, as shown in Table 2. The summary percentages are derived from majority vote, not from individual-level ratings.

**Table 2.** Prioritization results of 30 features.

Prioritization category	Final ratings, n (%)	Unanimous vote (4/4), n (%)	Majority vote (3/4), n (%)
Must have	11 (37)	8 (27)	3 (10)
Should have	4 (13)	1 (3)	3 (10)
Could have	6 (20)	3 (10)	3 (10)
Won’t have	0 (0)	0 (0)	0 (0)
Split/distributed	9 (30)	—^a^	—
Should-could	4 (44)^b^	—	—
Must-should	3 (33)^b^	—	—
Must-should-could	2 (22)^b^	—	—

a Not applicable.

b Denominator for split/distributed categories is 9.

Table 3 details the ranking changes and what features were implemented. Of the 30 features, 14 were implemented. There were 10 features that received all “must have” rankings; of those features, 6 were ultimately implemented into the final system; features 12, 15, 20, and 26 received all “must have” rankings, but were not developed. Of the remaining features that were not implemented but still received at least 2 “must have” rankings, feature 19 had 3 “must have” rankings and features 2 and 7 each had 2 “must have” rankings.

Descriptive analysis revealed 4 key MoSCoW insights (Table 4).

**Table 3.** Initial and final feature rankings and resulting implementation decision.

Feature number	Feature description	Initial ranking by participant				Final ranking by participant				Implemented
		P1	P2	P3	P4	P1	P2	P3	P4	
1	Ability to test rule results in the portal	M^a^	M	M	S^b^	S	C^c^	S	S	No
2	Designate which DAs^d^ are relevant by clinical specialty (or clinical departments)	M	NM^e^	M	M	S	S	M	M	No
3	Dynamic data dictionary of what DA entails	NM	NM	NM	NM	S	S	C	S	No
4	Explicit process for controlling import and validation of new artifacts (no discussion–no changes)	M	M	M	M	M	M	M	M	Yes
5	Indicate local SME^f^/clinical owner of DA content	M	M	M	M	M	M	M	M	Yes
6	Indicator that the DA is appropriate for routing to the patient portal	M	NM	NM	NM	M	C	S	S	Yes
7	Preview externally linked content to validate URLs	M	NM	NM	NM	M	S	M	S	No
8	Set DA favorites or bookmarks	S	S	NM	NM	S	S	C	M	No
9	Metadata detail of specialty that requested the rule	S	NM	C	S	S	C	C	S	No
10	Metadata on data fields that apply to CQL^g^ rules	NM	NM	NM	NM	C	C	S	C	No
11	Autocompletion suggestions when typing criteria	NM	NM	NM	S	M	S	S	M	Yes
12	Identify rules using older versions of dependencies	NM	NM	NM	NM	M	M	M	M	No
13	SME details	NM	NM	NM	S	C	W	C	C	Yes
14	Authoring organization details	S	NM	C	S	S	S	S	S	Yes
15	Versioning reports	M	M	M	NR^h^	M	M	M	NR	No
16	Ability to search by free text	M	M	M	NR	M	M	M	NR	Yes
17	Display datasets and logic statements used by DA in search results	NM	NM	NM	NM	C	C	C	C	No
18	Display last time DA was used and DA developer information in search results	S	NM	NM	NM	S	C	S	S	Yes
19	Suggest DAs recently used when searching	S	M	C	NM	M	M	S	M	No
20	Commonly used reports	M	M	M	M	M	M	M	M	No
21	System to manage who made or vetted the rule or why	NM	NM	M	M	M	M	M	M	Yes
22	Information about governing bodies proposing and approving rules	S	NM	NM	NM	S	C	C	C	Yes
23	Clinical experts sign off on the rules	M	M	NM	M	M	M	M	M	Yes
24	Operational leadership sign-off rules for operational stuff	NM	NM	NM	C	S	S	C	C	No
25	High upper governance approval of terminology	NM	NM	NM	NM	C	C	C	C	Yes
26	Schedule/reminders to review rules, guidelines, and evidence	M	M	M	M	M	M	M	M	No
27	Track information about person testing the rules	S	C	C	S	S	C	C	S	No
28	Dedicated SEDANS^i^ testing environment for CQL rules	NM	NM	NM	S	S	C	C	S	No
29	Track when the rule was made	M	M	M	M	M	M	M	M	Yes
30	A list by institution of specialists with clinical expertise who could be consulted for threshold values	NM	NM	NM	C	C	C	C	C	Yes

a M: must have.

b S: should have.

c C: could have.

d DA: decision aid.

e NM: not moved.

f SME: subject matter expert.

g CQL: clinical quality language.

h NR: not ranked.

i SEDANS: Shared Decision Aid Navigator System.

**Table 4.** Descriptive qualitative insights from the MoSCoW (must have, should have, could have, won’t have) transcripts.

Key insight	Summary	Illustrative quotes
Interpretations of feature descriptions may vary	Participants often revised feature rankings after clarification of the feature description revealed differences between their mental model and intended design.	“In practice, where else can we test the rules, if outside of the production environment?” [P4] “Would there be a benefit to testing this in the portal, outside of it…looking prettier, being web-based, is there any clinical advantages testing in the portal?” [P2]
Group discussion generated suggestions for alternative design solutions	Clarifying feature goals led participants to propose alternative designs that better addressed the underlying user need.	“I would imagine you probably can’t from the portal, as efficiently, right? I would imagine testing from a portal will be difficult, more difficult than testing from an authoring tool?” [P4] “The interesting thing about this is that maintaining and keeping it updated, having that kind of thing expire and let someone know that it needs to be verified, it’s going to be important. If this data lives here and that person becomes no longer the person who’s, you know, the owner, if it doesn’t get updated here no one’s going to know.” [P2]
Group discussion identified implementation considerations	Discussion surfaced maintenance and governance requirements that could affect long-term feature viability.	“In real life I feel this gets a little redundant and annoying, and you have to have operational sign off for any new thing. But usually, the subject matter expert that’s involved is usually connected to the operations people in real life right, so say we’re doing a new rule for breast cancer screening, usually the person, usually the SME, is someone Operations has vetted and blessed as the person to talk to as your consultant for designing the role, you know.” [P4] “Yeah, I feel like, I feel like Governance should approve like the concept or the intent, but not the gritty details like that.” [P3]
Participants considered differences in real-world use vs idealized use when ranking features	Participants deprioritized features that seemed useful in theory but impractical or burdensome in real-world implementation.	“As soon as people start getting automated reports that they have to respond to and take responsibility, then they make sure that it’s maintained correctly in this system. ‘Oh, you’re not in charge of this? Well, you better find out who it is because you’re getting the e-mail again next month.’” [P2] “This is tough, man. Once again, like one of those real-life things. I feel like it’s the SME that makes the call on that stuff.” [P3]

## Discussion

### Principal Findings

Our experience with the MoSCoW method was positive: it was easy to execute, intuitive for participants, and enabled prioritization of all 30 features in 74 minutes, indicating feasibility in this case. This aligns with Hatton et al [17], who found that simple prioritization techniques facilitate timely, expert-driven input with minimal burden. Table 4 clearly illustrates that not all the features that were deemed valuable (ie, received at least 2 “must have” rankings) were ultimately implemented. However, the information that was received in the MoSCoW session informed our research team of different perspectives and considerations, including considerations of other institutions. This is valuable because so often in the process of UCD sprints, many features are generated during the brainstorming phase [5]. Therefore, there is a limitation to end-user engagement when conducting discovery work for needs and requirements. Due to a potential large number of suggestions, designers and researchers need a process to identify which features should be developed. To address this, the DEAN research team applied a supplemental consensus process incorporating clinical, technical, cost, and complexity considerations.

Ultimately, the prioritization results informed actual DEAN design decisions, although not in a one-to-one fashion, that is, a “must have” ranking did not equal integration into the administrative portal. However, prioritization results were one strand of evidence that informed the team decision. Discussions during the MoSCoW session informed the research team, which was ultimately responsible for making final decisions on what features would be included in the administrative portal. This is a key point in our design process. Our team strengthened the MoSCoW prioritization by combining it with T-shirt sizing, a lightweight method for estimating implementation effort. In a multidisciplinary team, stakeholders first classified requirements as “must have,” “should have,” “could have,” or “won’t have” using the MoSCoW framework. Our technical lead (AS) then assigned a development and programming effort estimate (eg, extra small, small, medium, large, extra large) to each requirement based on the expertise of the developers assigned to this project. Considering both priority and effort enabled our multidisciplinary team to identify high-value needs and requirements to determine features that can be implemented quickly, while recognizing high-effort items that may require additional planning or resources. This combination supported transparent decision-making, balanced collaborator needs with development constraints, and facilitated decision-making during our prioritization process.

The interactive group format was critical for establishing shared understanding. HIT professionals often interpreted feature descriptions differently than intended. Group discussion enabled real-time clarification, supported less vocal participants, and improved prioritization accuracy. Beyond clarification, discussion yielded alternative design approaches and surfaced key implementation considerations, enriching the UCD process with qualitative insights unlikely to emerge from individual ratings.

We recommend using clear, concise feature descriptions, explicitly stating the pain point each feature addresses, and incorporating opportunities for clarification, particularly in live settings where domain experts can translate terminology. Evaluation of potential feature descriptions for use during the MoSCoW method may further improve clarity, especially for asynchronous formats.

### Comparison to Prior Work

Our findings are consistent with prior reports that MoSCoW is valued for its simplicity, accessibility, and ability to support rapid stakeholder engagement. Requirement prioritization reviews have identified MoSCoW as one of the most used techniques because it is easy to learn and can be implemented without extensive training or specialized analytic methods [10,11]. Similar to previous software development studies, participants in our session were able to rapidly classify features and complete prioritization within a single meeting, suggesting that the method can efficiently support early design decisions in health informatics settings. These observations extend prior work by demonstrating how MoSCoW can be incorporated within a UCD workflow involving HIT professionals rather than traditional software development teams [10,11,16].

Our results also reinforce concerns previously noted regarding category saturation and limited prioritization granularity. Consistent with the observations of Vijayakumar et al [18], a substantial proportion of features were classified as “must have,” while no features received a final “won’t have” designation. This pattern may reflect the tendency of stakeholders to focus on perceived value rather than resource constraints when evaluating proposed functionality. The presence of split ratings and subsequent discussion-based rating changes further suggests that MoSCoW alone may be insufficient for distinguishing among high-priority requirements when multiple features appear similarly important. As reported in comparative evaluations of prioritization techniques, supplemental consensus-building processes may be necessary to achieve more actionable rankings in feature-rich projects [11,16,18]. Future work should assess structured consensus steps to resolve ties.

A key contribution of this study is the observation that the value of MoSCoW extended beyond the prioritization outcomes themselves. Previous literature has primarily evaluated MoSCoW based on its ability to generate ranked requirements [11,16]. In contrast, our findings highlight how group discussion surrounding feature ratings generated design alternatives, surfaced implementation constraints, and revealed differing assumptions about real-world workflows. These observations align with UCD principles emphasizing stakeholder dialogue as a mechanism for understanding sociotechnical contexts and uncovering latent requirements [1]. The rating changes observed during discussion suggest that HIT professional interaction may be as valuable as the final rankings, particularly in HIT projects where implementation success depends on organizational workflows, governance structures, and maintenance considerations [1,17].

### Strengths and Limitations

There were several strengths of this study. First, the study was conducted in a real-world UCD workflow in an active HIT development process. This potentially increases the practical relevance and contextual validity of the single-case findings. Second, the project leveraged the expertise of HIT professionals and therefore grounds the work in existing contexts with unique implementation considerations.

This study also has several limitations. First, group dynamics may have influenced ratings. We did not assess participants’ perceived comfort or freedom to express their opinions during the structured interaction. Study staff encouraged HIT professionals to be as transparent and open as possible. As a result, we cannot conclude that all participants felt equally able to openly share their perspectives. In future work, measures should be collected on comfort and satisfaction of the participants’ interaction. Second, while the method worked well for 30 features, scalability to substantially larger feature sets remains uncertain.

### Future Directions

Next steps in evaluating the MoSCoW plus UCD process should include comparison against other prioritization methods. This potential work could be important to health informaticians and designers if the results support the selection of a prioritization process based on resources and skill sets available. Another area of interest is evaluating the relative merit of independent ratings compared to the discussion between HIT professionals, as observed in this study. The objective of this work would be to determine how much, and in what ways, discussion among experts impacts the prioritization outcome, potential consensus formation, and ultimate feature implementation. Findings in this area could potentially shape a distinction for MoSCoW as a stand-alone ranking approach or a framework for collaborator engagement and deliberation within UCD sessions.

### Conclusions

We found the MoSCoW method to be an understandable approach for HIT professionals that was rapid and intuitive. The process provided structured support for research team decision-making throughout a complex UCD software development project in health informatics. The approach appeared to be potentially feasible and a practical way to involve potential end users in the feature prioritization process, while also gaining insightful input to inform the multidisciplinary research team’s design decisions.

## Supplementary material

10.2196/30427 Multimedia Appendix 1 Feature descriptions and health information technology ratings during the user-centered design MoSCoW (must have, should have, could have, or won’t have) session.

## Abbreviations

DEAN: Decision Aid Navigator
HIT: health information technology
MoSCoW: must have, should have, could have, won't have
UCD: user-centered design

## References

[1] Schulze AN (2001). User-centered design for information professionals. J Educ Libr Inf Sci.

[2] Abras C, Maloney-Krichmar D, Preece J, Bainbridge WS (2004). Encyclopedia of Human-Computer Interaction.

[3] Morse B, Soares A, Ytell K (2023). Co-design of the Transgender Health Information Resource: web-based participatory design. J Particip Med.

[4] Morse B, Reale C, Nguyen AT (2025). Clinician needs and requirements for a Decision Aid Navigator: qualitative study. JMIR Hum Factors.

[5] van Velsen L, Ludden G, Grünloh C (2022). The limitations of user-and human-centered design in an eHealth context and how to move beyond them. J Med Internet Res.

[6] The DSDM Agile Project Framework: Handbook.

[7] Niculescu A (2024). MoSCoW prioritization: a comprehensive guide to effective project requirements management. HyperSense Software.

[8] O’Connor SR, Wilson V, Brown D, Cleland I, McCance TV (2024). Development and optimisation of a mobile app (iMPAKT) for improving person-centred practice in healthcare settings: a multi-methods evaluation study. Digit Health.

[9] Ahmad KS, Ahmad N, Tahir H, Khan S (2017). 2017 International Conference on Intelligent Computing, Instrumentation and Control Technologies (ICICICT).

[10] Borhan NH, Zulzalil H, Hassan S, Hayati N, Ali M (2022). Requirements prioritization in Agile projects: from experts’ perspectives. J Theor Appl Inf Technol.

[11] Bukhsh FA, Bukhsh ZA, Daneva M (2020). A systematic literature review on requirement prioritization techniques and their empirical evaluation. Comput Stand Interfaces.

[12] Clegg D, Barker R (1994). Case Method Fast-Track: A Rad Approach.

[13] Mandel JC, Kreda DA, Mandl KD, Kohane IS, Ramoni RB (2016). SMART on FHIR: a standards-based, interoperable apps platform for electronic health records. J Am Med Inform Assoc.

[14] Curran RL, Kukhareva PV, Taft T (2020). Integrated displays to improve chronic disease management in ambulatory care: a SMART on FHIR application informed by mixed-methods user testing. J Am Med Inform Assoc.

[15] Rindal DB, Pasumarthi DP, Thirumalai V (2023). Clinical decision support to reduce opioid prescriptions for dental extractions using SMART on FHIR: implementation report. JMIR Med Inform.

[16] Hudaib A, Masadeh R, Qasem MH, Alzaqebah A (2018). Requirements prioritization techniques comparison. Mod Appl Sci.

[17] Hatton S, Hainaut JL, Rundensteiner EA, Kirchberg M (2007). Advances in Conceptual Modeling – Foundations and Applications.

[18] Vijayakumar S, Prasad KK, Holla MR (2024). Assessing the effectiveness of MoSCoW prioritization in software development: a holistic analysis across methodologies. EAI Endorsed Trans Internet Things.

[19] Zoom.

[20] Morse B, Anstett T, Mistry N (2023). User-centered design to reduce inappropriate blood transfusion orders. Appl Clin Inform.

[21] Mural.

[22] Morse B, Soares A, Kwan BM (2023). A transgender health information resource: participatory design study. JMIR Hum Factors.

[23] Yin RK (2018). Case Study Research and Applications: Design and Methods.

[24] Stelter R (2015). “I tried so many diets, now I want to do it differently” - a single case study on coaching for weight loss. Int J Qual Stud Health Well-being.

[25] Crowe S, Cresswell K, Robertson A, Huby G, Avery A, Sheikh A (2011). The case study approach. BMC Med Res Methodol.

[26] Yin RK (2003). Applications of Case Study Research.

[27] Yin RK (1981). The case study crisis: some answers. Adm Sci Q.

[28] Hall S, Liebenberg L (2024). Qualitative description as an introductory method to qualitative research for master’s-level students and research trainees. Int J Qual Methods.

[29] Kim H, Sefcik JS, Bradway C (2017). Characteristics of qualitative descriptive studies: a systematic review. Res Nurs Health.

[30] Gale RC, Wu J, Erhardt T (2019). Comparison of rapid vs in-depth qualitative analytic methods from a process evaluation of academic detailing in the Veterans Health Administration. Implement Sci.

[31] Hamilton A Rapid turn-around qualitative research: strategic decision-making for rigor and feasibility. https://www.researchtalk.com/rapid-turn-around-qualitative-research-strategic-decision-making-for-rigor-and-feasibility.

[32] Vindrola-Padros C, Johnson GA (2020). Rapid techniques in qualitative research: a critical review of the literature. Qual Health Res.

[33] Kowalski CP, Nevedal AL, Finley EP (2024). Planning for and Assessing Rigor in Rapid Qualitative Analysis (PARRQA): a consensus-based framework for designing, conducting, and reporting. Implement Sci.

[34] Dodgson JE (2019). Reflexivity in qualitative research. J Hum Lact.

[35] Zairul M (2021). Can member check be verified in real time? Introducing ARC (asking, record, confirm) for member checking validation strategy in qualitative research. EJ.

