# Supplementary material for: Implementation of the MoSCoW Method and User-Centered Design to Prioritize Features for a Decision Aid Navigator (DEAN) System: Single-Case Qualitative Process Study
Source: JMIR Form Res. 2026 Oct 23;10:e30427. doi: 10.2196/30427 (PMC13694006; doi:10.2196/30427)
Supplement: Supplementary file 1 [file formative-v10-e30427-s001.docx]

**Appendix 1. HIT ratings during the UCD MoSCoW session**

|  |  | Initial | | | | Final | | | | Implemented |
| --- | --- | --- | --- | --- | --- | --- | --- | --- | --- | --- |
|  | Feature | PID1 | PID2 | PID3 | PID4 | PID1 | PID2 | PID3 | PID4 |  |
| 1 | Ability to test rule results in the portal | *M | M | M | **S | S | ***C | S | S | No |
| 2 | Designate which PDAs are relevant by clinical specialty (or clinical departments) | M | ****  NM | M | M | S | S | M | M | No |
| 3 | Dynamic data dictionary of what PDA entails | NM | NM | NM | NM | S | S | C | S | No |
| 4 | Explicit process for controlling import and validation of new artifacts (no discussion-no changes) | M | M | M | M | M | M | M | M | Yes. CQL files are parsed and version controlled. |
| 5 | Indicate local SME/clinical owner of PDA content | M | M | M | M | M | M | M | M | Yes |
| 6 | Indicator that the PDA is appropriate for routing to the patient portal. | M | NM | NM | NM | M | C | S | S | Yes. PDA states (edit, ready for review, approved, suspended, archived). |
| 7 | Preview externally linked content to validate URLs | M | NM | NM | NM | M | S | M | S | No. The user can click the link and open the DA in a browser. |
| 8 | Set PDA favorites or bookmarks | S | S | NM | NM | S | S | C | M | No. This was implemented in the Navigator app. |
| 9 | Metadata detail of specialty that requested the rule | S | NM | C | S | S | C | C | S | No |
| 10 | Metadata on data fields that apply to CQL rules | NM | NM | NM | NM | C | C | S | C | No. Users can access FHIR specification online. |
| 11 | Autocompletion suggestions when typing criteria | NM | NM | NM | S | M | S | S | M | Yes. Autocomplete for search. |
| 12 | Identify rules using older versions of dependencies | NM | NM | NM | NM | M | M | M | M | No. System shows only the latest version available. |
| 13 | SME details | NM | NM | NM | S | C | W | C | C | Yes |
| 14 | Authoring organization details | S | NM | C | S | S | S | S | S | Yes |
| 15 | Versioning reports | M | M | M | *****  NR | M | M | M | NR | No. Information is available in the logs. |
| 16 | Ability to search by free text | M | M | M | NR | M | M | M | NR | Yes |
| 17 | Display datasets and logic statements used by PDA in search results | NM | NM | NM | NM | C | C | C | C | No. Logic is processed by the navigator app. Users can view the logic of the CQL file. |
| 18 | Display last time PDA was used and PDA developer information in search results | S | NM | NM | NM | S | C | S | S | Yes. Available in dashboard. |
| 19 | Suggest PDAs recently used when searching | S | M | C | NM | M | M | S | M | No. |
| 20 | Commonly used reports | M | M | M | M | M | M | M | M | No |
| 21 | System to manage who made or vetted the rule or why | NM | NM | M | M | M | M | M | M | Yes. Upload FHIR Library resource with information about rule creator |
| 22 | Information about governing bodies proposing and approving rules | S | NM | NM | NM | S | C | C | C | Yes. Governance field |
| 23 | Clinical experts sign off on the rules | M | M | NM | M | M | M | M | M | Yes |
| 24 | Operational leadership sign off of rules for operational stuff | NM | NM | NM | C | S | S | C | C | No |
| 25 | High upper governance approval of terminology | NM | NM | NM | NM | C | C | C | C | Yes. Governance field. |
| 26 | Schedule/Reminders to review rules, guidelines and evidence | M | M | M | M | M | M | M | M | No |
| 27 | Track information about person testing the rules | S | C | C | S | S | C | C | S | No |
| 28 | Dedicated SEDANS testing environment for CQL rules | NM | NM | NM | S | S | C | C | S | No. CQL files are created and tested outside of the system and just uploaded. |
| 29 | Track when the rule was made | M | M | M | M | M | M | M | M | Yes. FHIR Library resource with information about rule creator. |
| 30 | A list by institution of specialists with clinical expertise who could be consulted for threshold values | NM | NM | NM | C | C | C | C | C | Yes. List of SMEs |

*Must

**Should

***Could

****Not moved

*****Not ranked
